# A serological biomarker of type I collagen degradation is related to a more severe, high neutrophilic, obese asthma subtype

**DOI:** 10.1186/s40733-022-00084-6

**Published:** 2022-04-13

**Authors:** Sarah Rank Rønnow, Jannie Marie Bülow Sand, Line Mærsk Staunstrup, Thomas Bahmer, Michael Wegmann, Lars Lunding, Janette Burgess, Klaus Rabe, Grith Lykke Sorensen, Oliver Fuchs, Erika V. Mutius, Gesine Hansen, Matthias Volkmar Kopp, Morten Karsdal, Diana Julie Leeming, Markus Weckmann, the ALLIANCE Study Group as part of the German Center of Lung Research (DZL)

**Affiliations:** 1 Nordic Bioscience A/S, Herlev, Denmark; 2 University of Southern Denmark, The Faculty of Health Science, Odense, Denmark; 3 University of Copenhagen, Health, Copenhagen, Denmark; 4 LungenClinic Grosshansdorf GmbH, Großhansdorf, Germany; 5 Airway Research Center North (ARCN), Member of the German Center for Lung Research (DZL), Großhansdorf, Germany; 6 Division of Asthma Mouse Model, Priority Area Asthma & Allergy, Leibniz-Center for Medicine and Biosciences Borstel, Borstel, Germany; 7 Department of Pathology and Medical Biology, Medical Biology Section, University Medical Center, Groningen, The Netherlands; 8 University Childrens Hospital, Inselspital Bern, Bern, Switzerland; 9 Dr. von Hauner Children’s Hospital, University Hospital Munich, Munich, Germany; 10 Comprehensive Pneumology Center Munich (CPC-M), Munich, Germany; 11 University Childrens Hospital, Department of Pediatric Pneumology, Allergology and Neonatology Hannover Medical School, Hannover, Germany; 12 Biomedical Research in Endstage and Obstructive Lung Disease Hannover (BREATH), Hannover, Germany; 13 Division of Pediatric Pneumology and Allergology, University Medical Center Schleswig-Holstein, Campus Centrum Lübeck, Lübeck, Germany

## Abstract

**Background:**

Asthma is a heterogeneous disease; therefore, biomarkers that can assist in the identification of subtypes and direct therapy are highly desirable. Asthma is a chronic inflammatory disease that leads to changes in the extracellular matrix (ECM) by matrix metalloproteinases (MMPs) degradation causing fragments of type I collagen that is released into circulation.

**Objective:**

Here, we asked if MMP-generated type I collagen (C1M) was associated with subtypes of asthma.

**Methods:**

C1M was serologically assessed at baseline in the adult participants of the All Age Asthma study (ALLIANCE) (*n* = 233), and in The Prospective Epidemiological Risk Factor study (PERF) (*n* = 283). In addition, C1M was assessed in mice sensitized to ovalbumin (OVA) and challenged with OVA aerosol. C1M was evaluated in mice with and without acute neutrophilic inflammation provoked by poly(cytidylic-inosinic) acid and mice treated with CP17, a peptide inhibiting neutrophil accumulation.

**Results:**

Serum C1M was significantly increased in asthmatics compared to healthy controls (*p* = 0.0005). We found the increased C1M levels in asthmatics were related to blood neutrophil and body mass index (BMI) in the ALLIANCE cohort, which was validated in the PERF cohort. When patients were stratified into obese (BMI > 30) asthmatics with high neutrophil levels and uncontrolled asthma, this group had a significant increase in C1M compared to normal-weight (BMI < 25) asthmatics with low neutrophil levels and controlled asthma (*p* = 0.0277). C1M was significantly elevated in OVA mice with acute neutrophilic inflammation compared to controls (*P* = 0.0002) and decreased in mice treated with an inhibitor of neutrophil infiltration (*p* = 0.047).

**Conclusion & clinical relevance:**

C1M holds the potential to identify a subtype of asthma that relates to severity, obesity, and high neutrophils. These data suggest that C1M is linked to a subtype of overall inflammation, not only derived from the lung. The link between C1M and neutrophils were further validated in *in vivo* model.

**Trial registration:**

(ALLIANCE, NCT02419274).

**Supplementary information:**

The online version contains supplementary material available at 10.1186/s40733-022-00084-6.

## Introduction

The most recent global estimate suggests that 334 million people have asthma worldwide [1]. Asthma is characterized by chronic inflammation of the airways associated with airway hyperresponsiveness leading to recurrent wheezing episodes, coughing, breathlessness, and chest tightness [2, 3]. The disease is heterogeneous with many phenotypes, but one of the most common endotypes is characterized by a high number of T helper type 2 (T_H_2) cells and a high sputum eosinophil number [4]. A range of biomarkers have been identified for the high T_H_2 asthma, but biomarkers for non-T_H_2 asthma endotype, also known as the non-eosinophilic asthma endotype, are lacking [5, 6]. However, sputum neutrophils, blood neutrophils, and IL-17 have been proposed as markers for the non-eosinophilic endotype [7–9].

An essential part of the pathogenesis of asthma is airway tissue remodeling [10]. The specific elements in airway remodeling and how they contribute clinically and functionally to asthma are poorly understood due to the disease heterogeneity [11, 12]. Airway remodeling may be determined by histological analysis or by an irreversible airway obstruction (post-bronchodilator forced expiratory volume in 1 s (FEV_1_) < 70%) [13, 14].

During airway tissue remodeling, subepithelial fibrosis leads to an altered extracellular matrix (ECM) content, contributing to asthma’s pathogenesis [15]. The existence of subepithelial fibrosis in asthmatic airways suggests an imbalance between matrix metalloproteinases (MMP) and their inhibitors [16]. Type I collagen is one of the most abundant collagens in the healthy lung, providing a structural framework for the airway wall [17–20]. Type I collagen plays a significant constitute in subepithelial fibrosis in asthmatic airways [17–20]. Due to increased levels of MMPs during inflammation in the asthmatic airways, the ECM quantity and quality are altered [21]. An excessive MMP level may lead to enhanced type I collagen degradation, resulting in increased production of a specific protein fragment known as C1M. C1M is released into the bloodstream and can be assessed as a biomarker of disease by a non-invasive ELISA and has been proposed as an inflammatory biomarker of soft tissue turnover in rheumatoid arthritis (RA) [22, 23].

Non-T_H_2 asthma is associated with more severe, high neutrophil levels and obesity [24–27]. Increased levels of neutrophils in the asthmatic airways may give rise to excessive production of neutrophil elastase and MMPs, possibly causing remodeling of the ECM [28, 29]. We, therefore, hypothesized that non-T_H_2 asthmatics would have elevated C1M levels in serum due to the increase of ECM remodeling in the airways. The biomarker C1M was measured serologically in the adult arm of the All Age Asthma (ALLIANCE) cohort to evaluate the utility of C1M to stratify non-T_H_2 subtypes of asthma. The findings were validated in the Prospective Epidemiological Risk Factor (PERF) study. C1M was furthermore investigated in an ovalbumin (OVA)-induced mouse model of allergic asthma to evaluate C1M as a translatable biomarker.

## Methods

The study design of the observational, multicenter adult arm of the ALL Age Asthma Cohort (ALLIANCE) of the German Center for Lung Research (DZL) (clinicaltrials.gov identifier NCT02419274) has previously been described in detail and approved by local ethics committees [30]. Briefly, the study presented here includes 233 participants, including 41 healthy controls, 86 severe asthmatics, and 106 mild-moderate asthmatics. The included asthmatic patients provided written informed consent and had an established diagnosis of asthma according to ERS/ATS 2014 guidelines. The study’s exclusion criteria were severe upper respiratory tract infection or severe exacerbation during the previous four weeks. Healthy controls did not have any pulmonary disease but could present with allergic rhinoconjunctivitis. Impulse Oscillometry assessed lung airway resistance. According to GINA guidelines, uncontrolled asthma was defined as daytime symptoms more than twice a week, any night waking due to asthma, rescue medication needed more than twice a week and any activity limitation due to asthma [31, 32]. PERF is an epidemiological study, including 5855 Danish postmenopausal women, that has previously been described in detail [33]. The study was approved by the Copenhagen County Scientific Ethics Committee (jr: KA 99,070 gm), and written informed consent was obtained from all participants. All women in PERF were matched to the Danish National Patient Register through a personal identification number (CPR-number) [34]. Asthmatic patients (*n* = 283) were extracted from the PERF study by using the WHO’s International Classification of Diseases 10 (ICD10: J45, J46). The studies were carried out in accordance with the principles in the Declaration of Helsinki.

### OVA-induced mouse model of allergic asthma

**Fig. 1:**
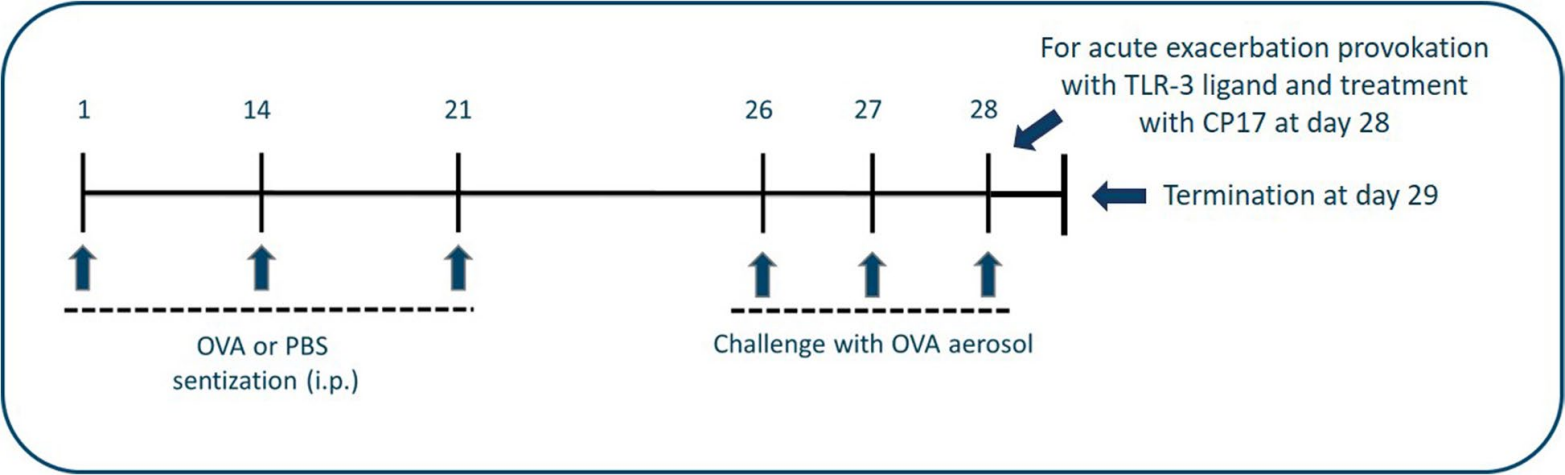
A schematic overview of the procedure in the OVA-induced asthma mouse model. Mice were sensitized to OVA (i.p.) on days 1, 14, and 21 and challenged with OVA aerosol on days 26, 27, and 28 to induce acute allergic airway inflammation. Acute neutrophilic inflammation were provoked by i.t. instillation of the TLR-3 ligand poly(cytidylic-inosinic) acid on day 28. A control group was sham sensitized to PBS and challenged with OVA aerosol. The mice were sacrificed on day 29

Experimentally induced allergic asthma in mice was performed as described previously [35, 36]. Briefly, 6–8 week old female C57BL/6 mice (Charles River, Sulzfeld, Germany) were sensitized to OVA (grade VI, Sigma, Deisenhofen, Germany) by intraperitoneal (i.p.) injections of 10 µg of OVA absorbed in 150 µg of aluminum hydroxide (Imject alum, Thermo, Rockford, Illinois, U.S.) on day 1, 14, and 21. The mice were challenged with OVA (OVA grade V, Sigma, Deisenhofen, Germany) aerosol (1% wt/vol in PBS) on day 26, 27, and 28 (*n* = 76) to induce acute allergic airway inflammation. The Toll-like receptor (TLR)-3 ligand poly(cytidylic-inosinic) (PolyIC) (Sigma-Aldrich, St Louise, Missouri, USA) provoked acute neutrophilic inflammation on day 28 (*n* = 20). Mice were treated with either CRP17, an active region of tumstatin inhibiting neutrophil influx, (*n* = 18), or a scrambled peptide (*n* = 18) [36]. A control group was sham sensitized to PBS and subsequently challenged with OVA aerosol (*n* = 20). The mice were sacrificed at day 29 by cervical dislocation under deep anesthesia **(**Fig. 1**)**. The mice were housed under specific pathogen-free conditions and received OVA free diet and water ad libitum. The animal ethics committee from the Department of State, Kiel, Germany, approved the animal study.

### Bronchoalveolar lavage

Via a tracheal canula the lungs were flushed with 1mL of sterile ice-cold PBS containing protease inhibitor (Complete, Roche, Basel, Switzerland). The obtained cells were counted using a light microscope (Leica, Wetzlar, Countess automated cell counter; Life Technologies, Darmstadt, Germany).

### Airway resistance and compliance

24 h after the last challenge with OVA, mice were anesthetized with ketamine, and xylazine and neuromuscular activity were blocked with pancuronium bromide (1 mg/kg) (Sigma, Deisenhofen, Germany). Tracheostomized mice were ventilated mechanically through a tracheal cannula that was attached to the FinePoint R/C system. Airflow and transpulmonary pressure were recorded continuously by using a Buxco FinePoint R/C system (DSI-Buxco Electronics, Sharon, CT, USA), calculating lung resistance (cm H_2_O/mL/s) and dynamic lung compliance (mL/cm H_2_O) in each breath cycle.

### Serological determination of type I collagen degradation

Degradation of type I collagen was assessed in human or mouse serum by a validated ELISA targeting a specific neoepitope of MMP-2,9,13 mediated degradation of type I collagen (C1M) (Nordic Bioscience, Herlev, Denmark) [22]. Measurements were performed in a blinded manner according to the manufacturer’s instructions. C1M was assessed previously in serum from participants in the PERF study [37].

### Statistical analysis

Basic demographics were compared using the Mann-Whitney U test, Kruskal-Wallis test, or chi-square test as appropriate after testing for normality. C1M levels or clinical parameters were compared between groups by the Kruskal-Wallis test, Mann-Whitney U test, or Spearman’s rank correlation as appropriate. MedCalc Statistical Software version 16.8.4, MedCalc Software bvba, Ostend, Belgium was used to perform all statistical analyses. Feature importance with respect to C1M levels was computed with a Random Forest model implemented through R (V. 3.5.1) with the caret R package (6.0.86). *P*-values > 0.05 was considered significant.

## Results

### Baseline characteristics

Basic demographics for participants with severe, mild-moderate asthma and healthy controls from the ALLIANCE study are listed in Table 1. Age, body mass index (BMI), and cigarette-smoking pack-years were significantly different between the three groups (*P* = 0.0001, *P* < 0.0001, and *P* = 0.005, respectively). Severe asthmatics had a significantly higher number of exacerbations and inhaled corticosteroids than mild-moderate asthmatics (*P* < 0.0001 and *P* < 0.0001, respectively). The group of severe asthmatics included 45% treated with oral corticosteroids, whereas none were treated with oral corticosteroids in the group of mild-moderate asthmatics. Basic demographics for asthmatic participants from the PERF study are listed in the online supplement e-Table 1.

**Table 1:** Basic demographics of the ALLIANCE cohort

	Severe asthma	Mild-moderate asthma	Healthy controls	*p*-value
N	**86**	**106**	**41**	
Age (yrs)	**56.2 ± 13.4**	**48.5 ± 13.0**	**46.3 ± 19.3**	***P*** ** = 0.0001** ^**a**^
Male	**40 (47)**	**45 (42)**	**24 (59)**	***P*** ** = 0.213** ^**c**^
Body-mass-index	**29.2 ± 6.9**	**26.8 ± 4.5**	**24.8 ± 3.9**	***P*** ** < 0.0001** ^**a**^
Current or former smoker ≥ 10PY	**23(27)**	**28(26)**	**3(7)**	***P*** ** = 0.030** ^**c**^
Pack years (yrs)	**6.9 ± 11.6**	**7.5 ± 13.6**	**1.4 ± 3.6**	***P*** ** = 0.005** ^**c**^
Blood Eosinophils	**0.43 ± 0.3**	**0.38 ± 0.4**	**0.17 ± 0.1**	***P*** ** < 0.0001** ^**a**^
Blood Neutrophils	**5.90 ± 2.8**	**4.00 ± 1.3**	**3.10 ± 1.0**	***P*** ** < 0.0001** ^**a**^
C1M ng/mL	**41.0 ± 44.1**	**31.0 ± 21.1**	**24.1 ± 12.4**	***P*** ** = 0.001** ^**a**^
Clinical variables				
FEV1 (L)	**2.2 ± 0.8**	**2.8 ± 0.7**	**3.7 ± 0.9**	***P*** ** < 0.0001** ^**a**^
FEV1 (% predicted)	**76.8 ± 21.4**	**94.0 ± 18.2**	**107.9 ± 10.1**	***P*** ** < 0.0001** ^**a**^
FVC (L)	**3.6 ± 1.0**	**4.1 ± 1.0**	**4.8 ± 1.1**	***P*** ** < 0.0001** ^**a**^
FVC (% predicted)	**97.8 ± 18.3**	**107.5 ± 15.5**	**113.6 ± 14.5**	***P*** ** < 0.0001** ^**a**^
FEV1/FVC	**0.60 ± 0.1**	**0.68 ± 0.1**	**0.76 ± 0.1**	***P*** ** < 0.0001** ^**a**^
sReff (kPa*s/L)	**1.8 ± 1.2**	**1.2 ± 0.8**	**0.8 ± 0.2**	***P*** ** < 0.0001** ^**a**^
sReff (% predicted)	**173.5 ± 118.9**	**116.2 ± 79.5**	**69.9 ± 20.1**	***P*** ** < 0.0001** ^**a**^
R5Hz (kPa/l/s)	**0.5 ± 0.2**	**0.5 ± 0.2**	**0.3 ± 0.1**	***P*** ** < 0.0001** ^**a**^
≥2 severe exacerbations in last 12 months	**48(56)**	**19(18)**	**n/a**	***P*** ** < 0.0001** ^**c**^
Treatments				
ICS (µg/day fluticasone)	**972.8 ± 492.6**	**302.0 ± 193.7**	**n/a**	***P*** ** < 0.0001** ^**b**^
OCS	**39 (45)**	**0**	**n/a**	

Data are shown as mean ± SD or number (%). *PY *pack-years, *FEV1 *post-bronchodilator forced expiratory volume in 1 s, *FVC* forced vital capacity, *sReff* body plethysmography, specific effective airway resistance, *R5Hz* impulse oscillometry, resistance at 5 Hz, *ICS* inhaled corticosteroids, *OCS* oral corticosteroids, *PY* pack years, *n/a* not applicable. Statistical significance was determined using Kruskal-Wallis^a^, Mann-Whitney U test^b^, or chi-squared test^c^ between the three groups

### Biomarker of type I collagen degradation is increased in asthmatic patients and correlates with airway resistance in the ALLIANCE cohort

**Fig. 2:**
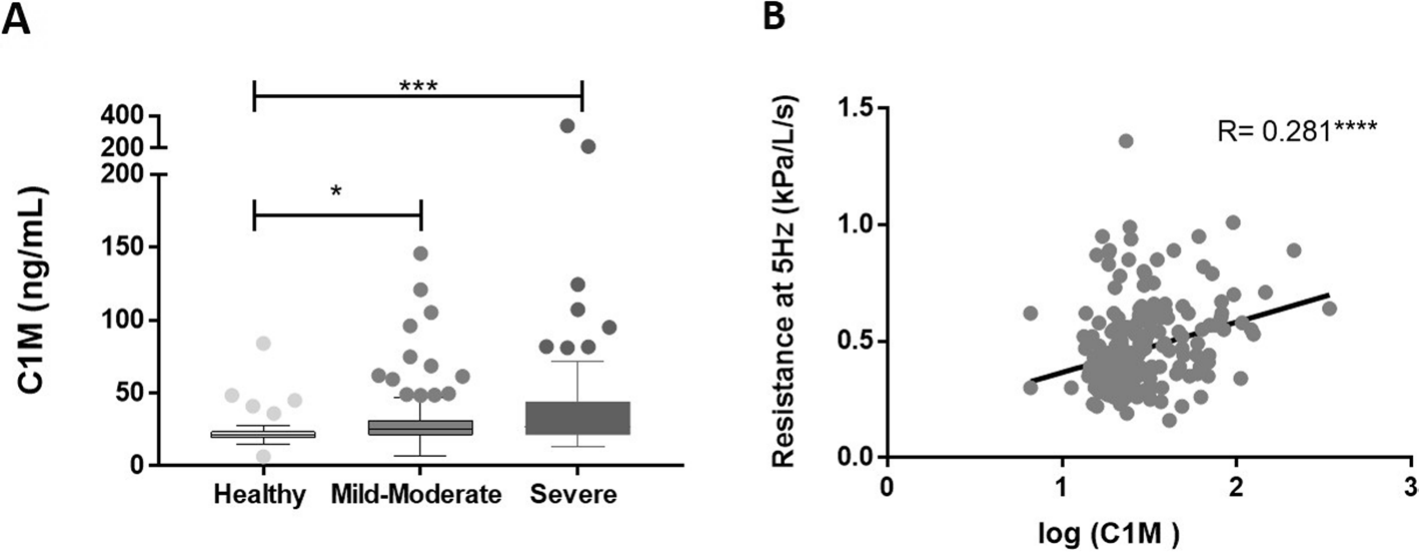
** A** Serum C1M levels were increased in severe asthmatics (*n* = 86) compared to healthy controls (*n* = 41) and mild-moderate asthmatics (*n* = 106) compared to healthy controls (*P* = 0.0005, *P* = 0.0075), respectively. Data are presented as a Tukey box plot and analyzed using the Kruskal-Wallis test using Dunn’s multiple comparisons. Asterisks indicate statistically significance: **p < 0.01, ***p < 0.001. **B** Correlation between serum log-transformed C1M level and airway resistance at 5 Hz. Data were analysed using spearman’s correlation (*r* = 0.281, *p* > 0.0001)

Serum levels of C1M were increased by 25.3% in severe asthmatic patients compared to healthy controls (*P* = 0.0005) and 17.7% in patients with mild-moderate asthma compared to healthy controls (*P* = 0.0075) **(**Fig. 2 A). No difference between mild-moderate and severe asthmatics was observed. Serum C1M levels were positively correlated with the airway resistance at 5 Hz (*r*_s_=0.281, *P* < 0.0001) (Fig. 2B). No association between C1M and FEV_1_ or forced vital capacity (FVC) was observed (data not shown).

### Type I collagen degradation is related to high blood neutrophil levels and obesity

**Fig. 3:**
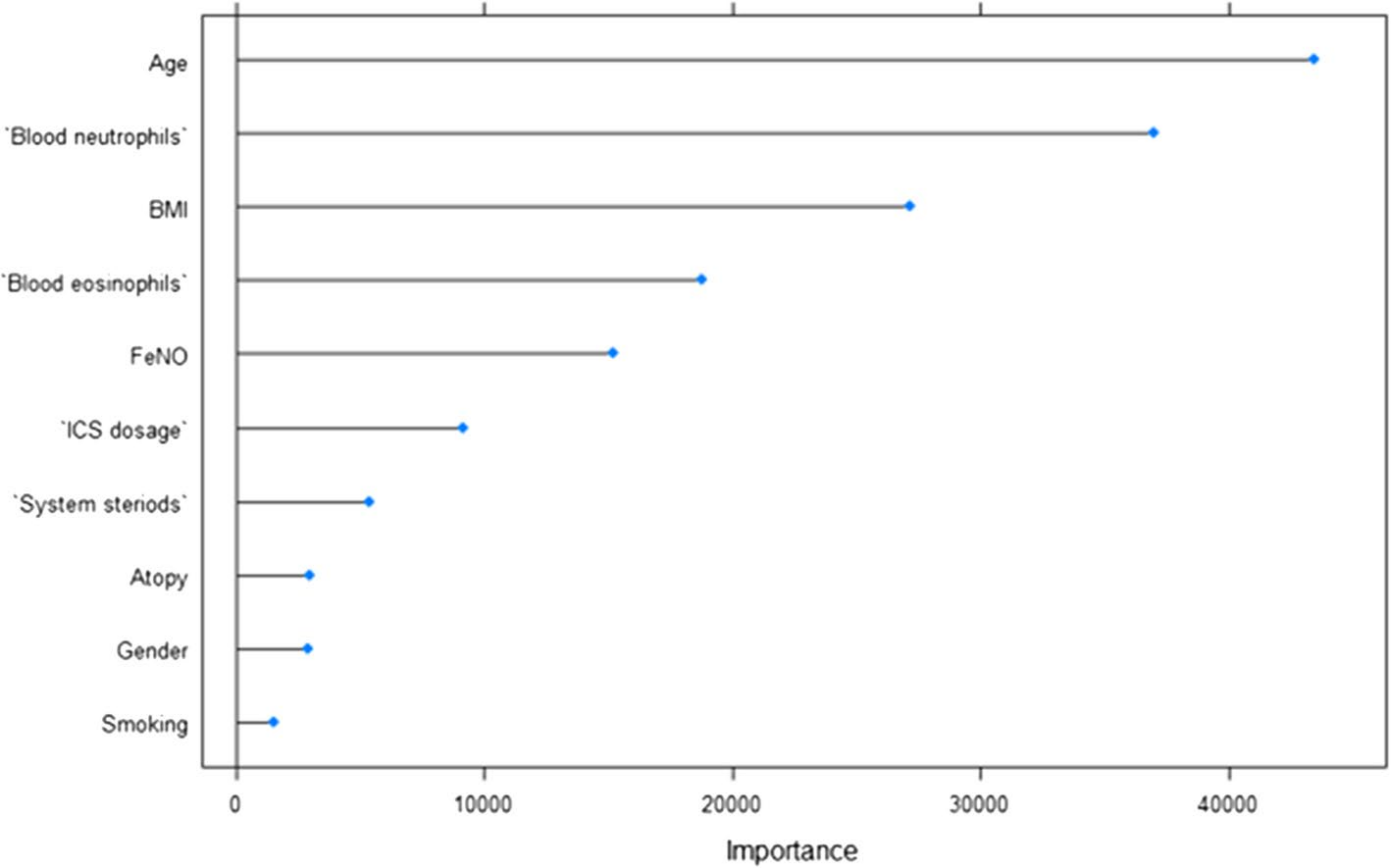
The relationship between BMI, age, smoking, allergic asthma, FeNO, the use of systemic steroids, inhaled corticosteroid dosage, blood neutrophils, and blood eosinophils on the levels of C1M in asthmatics were tested using a feature ranking using random forest. The top 3 most important attributes to C1M levels in the ALLIANCE cohort is Age, Blood neutrophils, and BMI

**Fig. 4:**
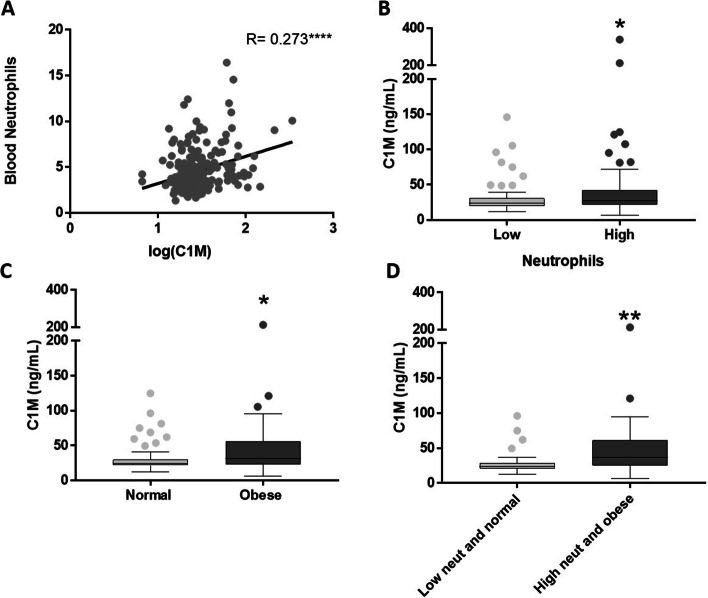
** A** Correlation between serum C1M level and blood neutrophils. Data were analysed using spearman’s correlation (*r* = 0.273, *p* < 0.0001). **B** Patients were stratified into high versus low blood neutrophils levels based on the median. C1M was significantly increased in patients with high neutrophil levels (*n* = 109) compared to low (*n* = 82) (*p* = 0.0154). **C** C1M was significantly increased in obese (BMI > 30) patients (*n* = 49) compared to normal-weight (BMI < 25) patients (*n* = 65) (*p* = 0.0137). **D** Obese asthmatics with high blood neutrophils (*n* = 33) had a significant increase in C1M compared to normal-weight asthmatics with low blood neutrophils (*n* = 36) (*p* = 0.0026). Data are presented as a Tukey box plot and analyzed using the Mann-Whitney test. Asterisks indicate statistically significance: *p < 0.05, **p < 0.01

Although C1M serum levels were found to increase in asthmatics compared to controls, the levels of C1M varied considerably. Therefore, we investigated the relationship between the increased C1M levels in asthmatics and the variables: BMI, age, smoking, allergic asthma status, fractional exhaled nitric oxide (FeNO), the use of systemic steroids, inhaled corticosteroid dosage, blood neutrophils, and blood eosinophils to determine which variables independently had the highest importance. A feature ranking using random forest showed the three variables with the highest independent importance for C1M levels in asthmatic patients were age, BMI, and blood neutrophils (Fig. 3). Using sputum neutrophils and eosinophils showed the same in the model as when blood neutrophils and eosinophils were used (data not shown). There was a weak but significant direct correlation observed between blood neutrophils and serum C1M levels in asthmatics (*r*_s_=0.273, *P* < 0.0001) (Fig. 4 A). No significant correlation was found between C1M and age, BMI or eosinophils nor between high versus low age based on the median (data not shown). Blood neutrophils were stratified into high versus low based on the median (4.2 × 10^3^/µL), and patients with high blood neutrophils had an increased C1M serum level as compared to patients with low blood neutrophils with median levels in high vs. low of 27.3 ± 40.3 vs. 23.8 ± 21.0 (*p* = 0.0154) (Fig. 4B). Stratification into obese (BMI > 30) and normal-weight (BMI < 25) asthmatic patients revealed an increased level of C1M in the obese subgroup with median levels in obese vs. normal-weight of 31.1 ± 34.9 vs. 24.8 ± 20.0 (*p* = 0.0137) (Fig. 4 C). Furthermore, obese asthmatic patients with high neutrophil levels had an increased C1M level compared to asthmatics with low neutrophil levels and normal-weight with median levels of 36.6 ± 38.7 vs. 23.6 ± 16.8, respectively (*p* = 0.0026) (Fig. 4D). These findings were confirmed in the PERF cohort where a significant correlation between blood neutrophils and C1M was observed (*r* = 0.214) (Fig. e1A), and patients with high blood neutrophil and obesity had significantly increased serum C1M levels compared to normal-weight asthmatic patients with low neutrophil levels (Fig. e1B-D). These data show that BMI and higher blood neutrophil counts are independent variables of the serum C1M levels increase in asthmatics.

### Type I collagen degradation is increased in a more severe, obese subtype of asthmatics with high blood neutrophil levels in the ALLIANCE cohort

**Fig. 5:**
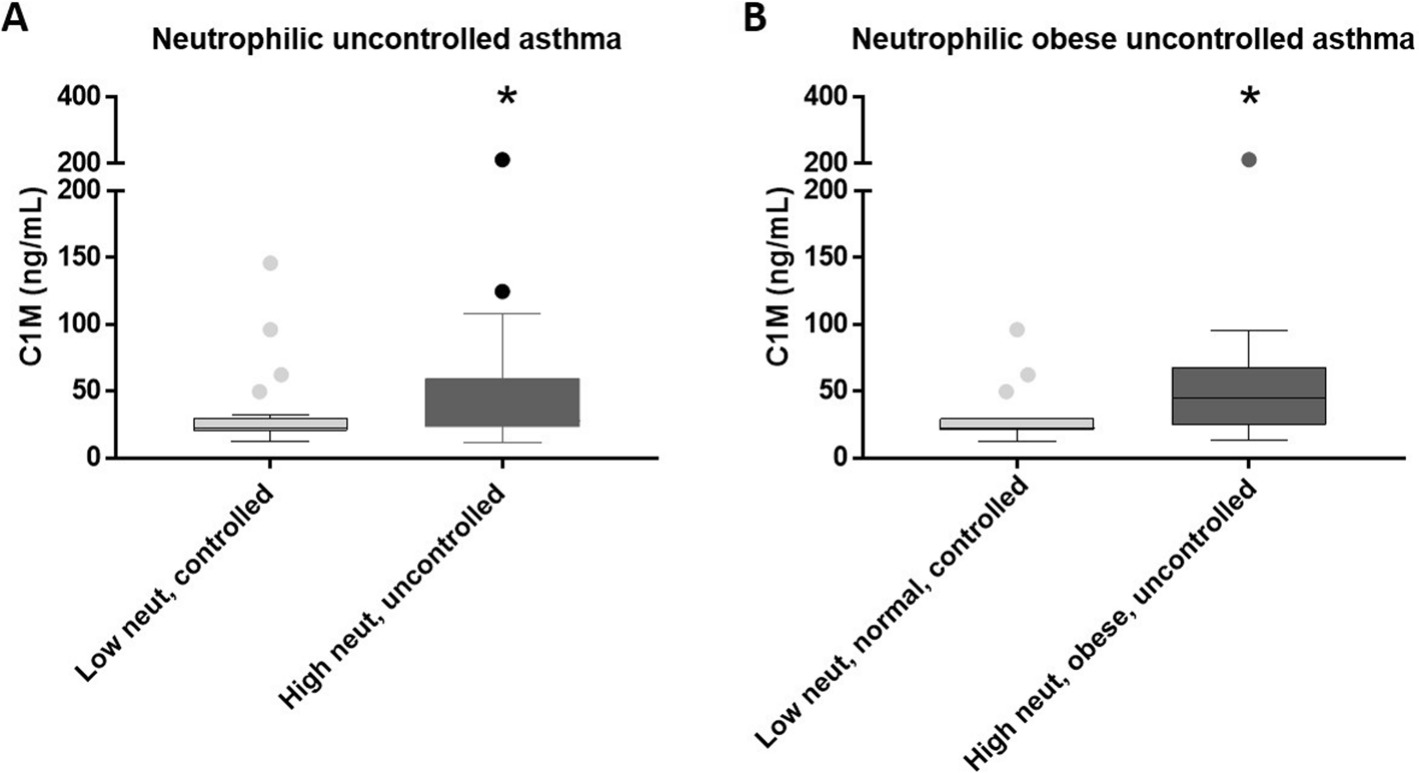
** A **Serum C1M levels were significantly increased in patients with uncontrolled asthma with a high blood neutrophil level (*n* = 53) compared to patients with controlled asthma and a low blood neutrophil level (*n* = 30) (*p* = 0.0387). **B** C1M was significantly increased in obese patients with high neutrophil levels and uncontrolled asthma (*n* = 20) compared to normal-weight patients with low neutrophil levels and controlled asthma (*n* = 15) (*p* = 0.0277). Data are presented as a Tukey box plot and analyzed using the Mann-Whitney test. Asterisks indicate statistically significance: *p < 0.05

The neutrophilic, obese asthma phenotype is often severe and presents with therapy refraction [26, 27]. To elucidate if the C1M high subtype of obese, high blood neutrophils asthma patients is related to asthma severity or a refractory response to therapy, patients were grouped into uncontrolled and controlled asthma (according to ATS/ERS guidelines). Patients with high neutrophil levels and uncontrolled asthma had an increased C1M level compared to patients with low neutrophils and controlled asthma with median levels of 27.8 ± 34.6 vs. 22.7 ± 26.9, respectively (*p* = 0.0387) (Fig. 5A). This was also seen when stratifying the patients into obese, high neutrophils, uncontrolled asthma compared to low neutrophils, normal-weight, controlled asthma with median levels of 45.1 ± 43.8 vs. 22.6 ± 21.9, respectively (*p* = 0.0277) (Fig. 5B).

### Type I collagen degradation is increased in an OVA-induced mouse model of allergic asthma and decreased by a peptide inhibiting neutrophil accumulation

**Fig. 6:**
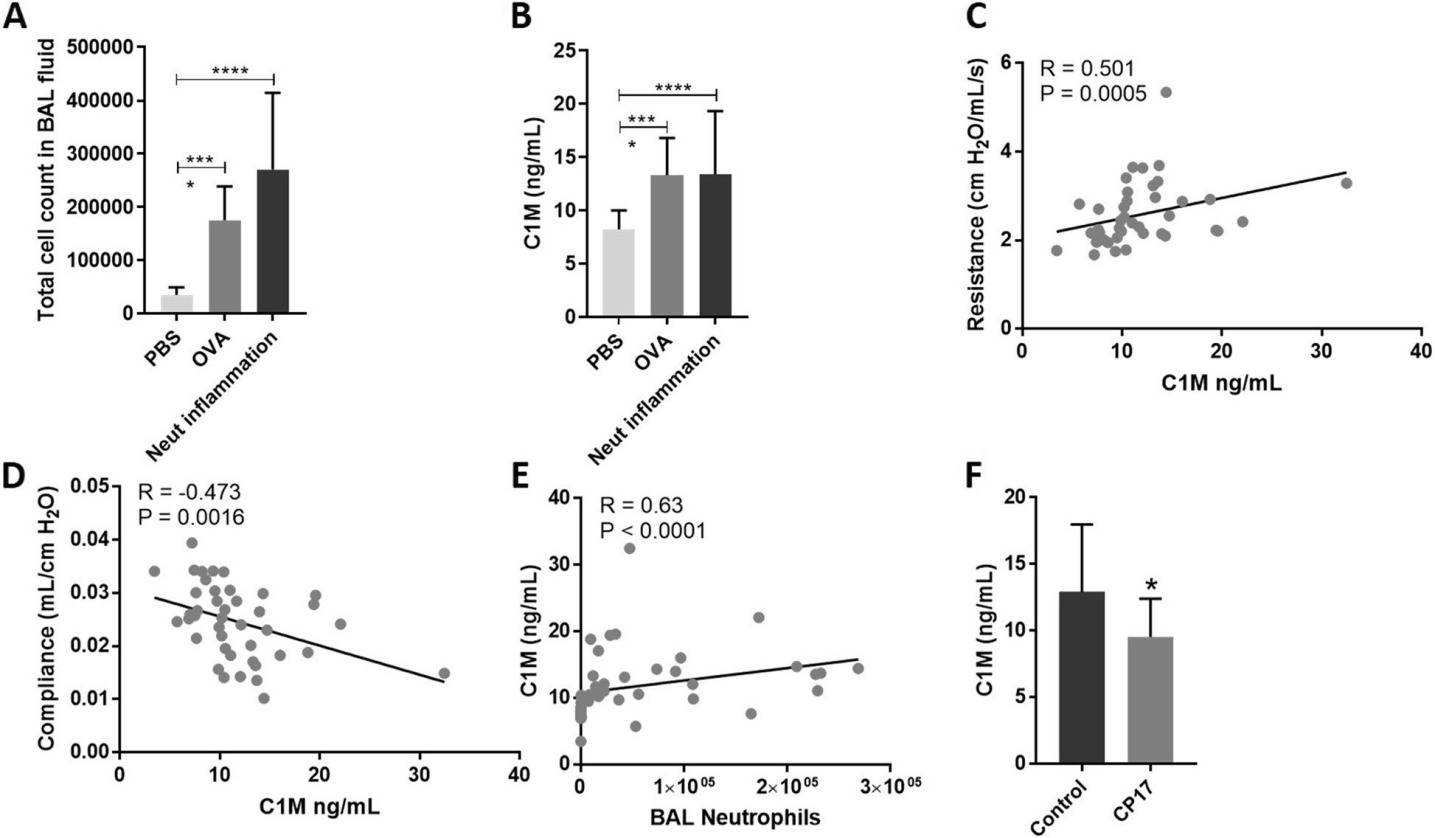
** A** Total cell count in the bronchoalveolar lavage (BAL) were significantly increased in OVA mice and acute neutrophilic inflammation compared to control (*P* < 0.0001, *P* < 0.0001), respectively **B** Serum C1M was significantly increased in serum from OVA mice and mice with an acute neutrophilic inflammation as compared to controls (*P* < 0.0001, *P* = 0.0002), respectively. **C** Correlation between serum C1M level and airway resistance (*r* = 0.501, *p* = 0.0005). **D** Correlation between serum C1M level and airway compliance (*r *= -0.473, *P* = 0.0016). **E** Correlation between serum C1M level and BAL neutrophil was not significant. **F** Serum C1M levels were decreased in OVA mice treated with CP17, a peptide inhibiting neutrophil accumulation, compared to OVA mice treated with a scrambled peptide (*p* = 0.047). Data are presented as bar graphs and analyzed using the Kruskal-Wallis test using Dunn’s multiple comparisons or the Mann-Whitney test. The correlation was analyzed using spearman’s correlation. Asterisks indicate statistically significance: *p < 0.05, ***p < 0.001 and ****p < 0.0001

Blood eosinophils and FeNO showed to be just below blood neutrophils, and BMI as independently important factors for the increased C1M level in asthmatics (Fig. 3). This may indicate that C1M is not specific for a non-T_H_2-asthma but related to a metabolic and more neutrophilic phenotype of asthma. To evaluate the neutrophilic component of C1M in asthma, we chose an OVA-induced mouse model of allergic asthma aggravated by PolyIC to mimic a neutrophil inflammation. A significant increase in the total cell count in the bronchoalveolar lavage (BAL) was found in OVA mice compared to PBS controls (*p* < 0.0001), and in mice with acute neutrophilic inflammation compared to controls (*p* < 0.0001). No difference in the total cell count of the BAL fluid was observed between OVA mice and mice with acute neutrophilic inflammation (Fig. 6 A). Serum levels of C1M were significantly increased in OVA mice compared to controls (*p* < 0.0001), and in OVA mice with acute neutrophilic inflammation compared to controls (*p* = 0.0002) but not in OVA mice with neutrophil inflammation as compared to without (Fig. 6B). Serum C1M levels were correlated with lung resistance and compliance in the OVA model (*r*_s_=0.501, *p* = 0.0005; *r*_s_=0.473, *p* = 0.0016; respectively) (Fig. 6 C-D). C1M serum levels correlated with the number of neutrophils in the BAL fluid (*r*_s_=0.63, *p* < 0.0001) (Fig. 6E). Treatment of challenged and exacerbated mice with CP17, which reduces neutrophil infiltration after PolyIC stimulation, showed a reduction in C1M levels compared to mice treated with a scrambled control peptide (*p* = 0.047) (Fig. 6 F).

## Discussion

Identifying asthma subtypes is of high importance to direct treatment and enable precision medicine. Here we investigated the relationship between type I collagen degradation (C1M) and subtypes of asthma. We measured C1M serologically in the ALLIANCE study, asthmatic participants in the PERF study, and an OVA-induced and PolyIC exacerbated, allergic asthma mouse model. We showed in two independent cohorts with asthmatic patients that C1M was related to high blood neutrophils and obesity, and further that C1M serum levels were associated with a loss of symptom control in these asthma patients. C1M levels were increased in mice with OVA-induced airway inflammation, but levels did not increase further with the addition of an experimental neutrophilic inflammation. Yet, C1M levels decreased when exacerbated animals were treated with a peptide inhibiting neutrophil infiltration, shining some light on the modalities of type I collagen degradation in asthma.

Chakir et al. showed by immunocytochemistry that type I collagen protein content was increased in the lungs from asthmatics compared to healthy controls [38]. Furthermore, Nomura et al. showed that type I procollagen C-terminal peptide (PICP), but not type I collagen C-terminal telopeptide (ICTP), levels were significantly higher in the sputum of asthmatics compared to healthy controls and that levels were inversely correlated with percentage FEV_1 _[17]. Both of these findings suggest that it is only type I collagen formation that is increased in asthmatics and not degradation. But in accordance with our study, Wolthers et al. demonstrated that budesonide was able to dose-dependently reduce ICTP, suggesting that collagen degradation may play a role in asthma as well [39]. This may suggest that it is the balance of collagen formation and degradation that is disturbed and contributes to the subepithelial fibrosis in asthmatic airways. In line with this hypothesis, it was demonstrated by Laliberté et al. that the balance between collagen synthesis and collagen degradation that exists in normal bronchial fibroblasts is disturbed in asthmatic bronchial fibroblast [40].

In our study, C1M serum levels were associated with blood neutrophils levels and obesity, which were both independent variables. It has already been shown that obesity can give rise to changes in the extracellular matrix explaining that the increase in C1M is higher in obese asthmatics compared to normal-weight asthmatics [41, 42]. The association of asthma with high levels of neutrophils and obesity has been investigated previously [26, 27, 43]. Moore et al. identified a subtype of obese females with late-onset severe asthma that also had increased neutrophil levels [6]. These data indicate that type I collagen degradation may be in part related to a more severe, therapy refractory and metabolic phenotype of asthma, such as the “late-onset female obese” phenotype.

It is challenging to investigate the severity of asthma since it is not a static term but changes over time due to disease flare-ups. Furthermore, asthma is a less progressive disease compared to IPF, where C1M has been shown to be able to identify disease progressors [44]. Chakir et al. showed that type I collagen stained more intense in patients with moderate-to-severe asthma compared to patients with mild asthma [38]. In addition, Nomura et al. demonstrated that PICP was significantly higher in patients having an exacerbation compared to the asthmatics experiencing no exacerbation [17]. We found C1M levels to be significantly increased in obese uncontrolled asthmatics with high neutrophil levels compared to normal-weight controlled asthmatics with low neutrophil levels. Together this suggests a complex mechanism in this asthma subtype with an underlying systemic effect caused by obesity, a high level of neutrophils, and increased destruction of the ECM and overall inflammation quantified by C1M, all causing worsening of the disease and thereby possibly uncontrolled asthma.

We partially replicated our human findings in a mouse model of asthma neutrophilic inflammation. Serum C1M levels were increased in OVA mice compared to controls, and C1M correlated both with airway resistance and compliance. These data indicate that type I collagen degradation also plays a role in the OVA-induced asthma mouse model. We saw a correlation between sputum neutrophils and C1M in this model, and treatment with CP17 [36]. an active peptide of tumstatin inhibiting neutrophil infiltration in the lung reduced C1M significantly compared to the scrambled peptide control. These findings suggest that there is an underlying relationship between neutrophil infiltration in the lung and C1M in this model. Finally, the presented data indicate that C1M may be used as a translational link between humans and mice for further investigations and drug discovery.

Further mechanistic research is necessary to understand the release of C1M in this asthmatic subtype, but during inflammation, neutrophils produce MMP-9 that can cleave type I collagen and generate the biomarker C1M [29, 45]. Furthermore, neutrophils play a part as a driving factor of chronic inflammation in COPD, possibly, explaining the increased C1M levels observed in COPD patients during disease progression [19, 46]. Besides, C1M has already been proposed as a marker for systemic inflammation in RA [23]. These data indicate that the increased levels of C1M in this asthma subtype might be explained by an overall inflammation created by obesity and the high neutrophil levels. The biomarker C1M might help to investigate the underlying mechanisms of this subtype and, in that way direct treatment.

### Limitation

Even though some of the results are validated in two independent cohorts, the link between obesity, high neutrophils, and uncontrolled asthma remains to be confirmed in a larger cohort. Also, the two cohorts used to investigate this relationship was were different in demographics. Especially because the PERF cohort only included elderly women. In addition, measuring a biomarker in the patient’s blood makes it non-invasive but also less tissue-specific making it difficult to determine if the high levels of type I collagen degradation is due to asthma or obesity since the groups had significantly different BMI.

## Conclusions

We found type I collagen degradation to be increased in asthmatic patients compared to healthy control and the increase to be associated with blood neutrophils and obesity in two independent cohorts. High levels of type I collagen degradation was associated with a subtype of obese asthmatics with high neutrophil levels and uncontrolled asthma. The involvement of neutrophil inflammation was supported by our OVA-induced and PolyIC aggravated asthma mouse model, as collagen type I degradation was significantly decreased with treatment with an inhibitor of neutrophil accumulation. These data indicate that type I collagen degradation might play a part in the pathogenesis of metabolic and neutrophil subtypes of asthma and that C1M may be used as a translational biomarker to direct therapy in these specific asthma subtypes.

## Supplementary Information

**Additional file 1: Table e1. **Basic demographics of the PERF cohort.** E-Figure1.** A) Correlation betweenserum C1M level and blood neutrophils. Data were analysed using spearman’scorrelation (*r* = 0.214). B) Patients were stratified into highversus low percentage blood neutrophils levels based on the median. C1M wassignificantly increased in patients with high neutrophil levels (*n* = 122) compared to low (*n* = 128) (*p* = 0.0130). C) C1Mwas significantly increased in obese (BMI>30) patients (*n* = 60) compared to normal-weight (BMI<25) patients (*n* = 85) (*p* < 0.0001). D) Obeseasthmatics with high blood neutrophils (*n*= 29) had a significant increase in C1M compared to normal-weight asthmaticswith low blood neutrophils (*n* = 39) (*p* < 0.0001). Data arepresented as a Tukey box plot and analyzed using the Mann-Whitney test.Asterisks indicate statistically significance: *p < 0.05, ****p < 0.0001.

## Data Availability

The data that support the findings of this study are available on request from the corresponding author. The data are not publicly available due to privacy or ethical restrictions.

## Abbreviations

ALLIANCE: All Age Asthma cohort
BAL: Bronchoalveolar lavage
BMI: Body mass index
C1M: MMP generated type I collagen
ECM: Extracellular matrix
FeNO: Fractional exhaled nitric oxide
FEV_1_: Forced expiratory volume in 1 second
FVC: Forced vital capacity
ICTP: Type I collagen C-terminal telopeptide
MMP: Matrix metalloproteinases
OVA: Ovalbumin
PERF: The Prospective Epidemiological Risk Factor study
PICP: Type I procollagen C-terminal peptide
PolyIC: Poly(cytidylic-inosinic)
RA: Rheumatoid arthritis
T_H_2: T helper type 2
TLR: Toll-like receptor
