# Supplementary material for: A serological biomarker of type I collagen degradation is related to a more severe, high neutrophilic, obese asthma subtype
Source: Asthma Res Pract. 2022 Apr 13;8:2. doi: 10.1186/s40733-022-00084-6 (PMC9006548; doi:10.1186/s40733-022-00084-6)
Supplement: Supplementary file 1 — Additional file 1: Table e1. Basic demographics of the PERF cohort. E-Figure1. A) Correlation betweenserum C1M level and blood neutrophils. Data were analysed using spearman’scorrelation (r = 0.214). B) Patients were stratified into highversus low percentage blood neutrophils levels based on the median. C1M wassignificantly increased in patients with high neutrophil levels (n = 122) compared to low (n = 128) (p = 0.0130). C) C1Mwas significantly increased in obese (BMI>30) patients (n = 60) compared to normal-weight (BMI<25) patients (n = 85) (p < 0.0001). D) Obeseasthmatics with high blood neutrophils (n= 29) had a significant increase in C1M compared to normal-weight asthmaticswith low blood neutrophils (n = 39) (p < 0.0001). Data arepresented as a Tukey box plot and analyzed using the Mann-Whitney test.Asterisks indicate statistically significance: *p < 0.05, ****p < 0.0001. [file 40733_2022_84_MOESM1_ESM.zip › Table e1.docx]

**Table e1:** Basic demographics of the PERF cohort

|  | Asthmatics from the PERF study |
| --- | --- |
| **N** | **250** |
| **Age (yr)** | **70.1 ± 6.4** |
| **Male (%)** | **0 (0)** |
| **BMI** | **26.9 ± 4.4** |
| **Current or former smoker (%)** | **40 (111)** |
| **Pack years** | **>146 packs/year (70)**  **between 146-292 packs/year (69)**  **<292 packs/year (12)** |

Data are shown as mean ±SD or number (%).

**E-Figure 1:** **A)** Correlation between serum C1M level and blood neutrophils. Data were analysed using spearman’s correlation (r=0.214). **B)** Patients were stratified into high versus low percentage blood neutrophils levels based on the median. C1M was significantly increased in patients with high neutrophil levels (n=122) compared to low (n=128) (p=0.0130). **C)** C1M was significantly increased in obese (BMI>30) patients (n=60) compared to normal-weight (BMI<25) patients (n=85) (p<0.0001). **D)** Obese asthmatics with high blood neutrophils (n=29) had a significant increase in C1M compared to normal-weight asthmatics with low blood neutrophils (n=39) (p<0.0001). Data are presented as a Tukey box plot and analyzed using the Mann-Whitney test. Asterisks indicate statistically significance: *p<0.05, ****p<0.0001
