# Supplementary figures and images for: A serological biomarker of type I collagen degradation is related to a more severe, high neutrophilic, obese asthma subtype
Source: Asthma Res Pract. 2022 Apr 13;8:2. doi: 10.1186/s40733-022-00084-6 (PMC9006548; doi:10.1186/s40733-022-00084-6)

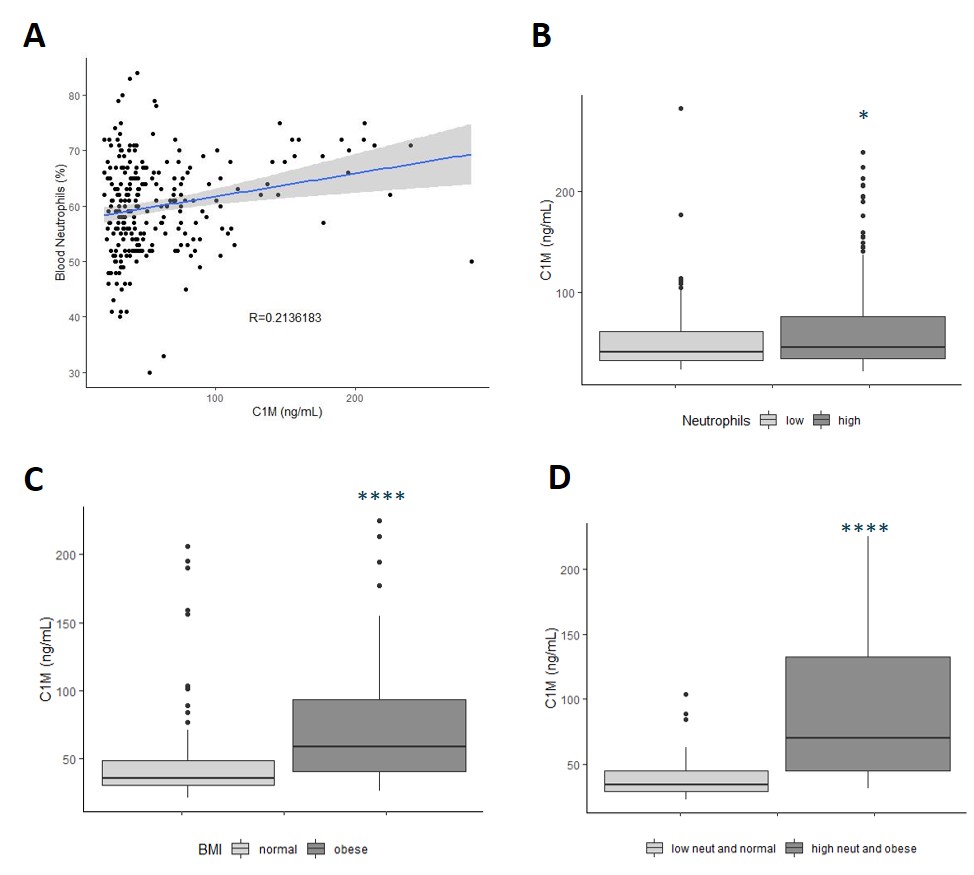

Supplement: Supplementary file 1 — Additional file 1: Table e1. Basic demographics of the PERF cohort. E-Figure1. A) Correlation betweenserum C1M level and blood neutrophils. Data were analysed using spearman’scorrelation (r = 0.214). B) Patients were stratified into highversus low percentage blood neutrophils levels based on the median. C1M wassignificantly increased in patients with high neutrophil levels (n = 122) compared to low (n = 128) (p = 0.0130). C) C1Mwas significantly increased in obese (BMI>30) patients (n = 60) compared to normal-weight (BMI<25) patients (n = 85) (p < 0.0001). D) Obeseasthmatics with high blood neutrophils (n= 29) had a significant increase in C1M compared to normal-weight asthmaticswith low blood neutrophils (n = 39) (p < 0.0001). Data arepresented as a Tukey box plot and analyzed using the Mann-Whitney test.Asterisks indicate statistically significance: *p < 0.05, ****p < 0.0001. [file 40733_2022_84_MOESM1_ESM.zip › Figure e1.jpg]
